# Dynamics of the mouse brain cortical synaptic proteome during postnatal brain development

**DOI:** 10.1038/srep35456

**Published:** 2016-10-17

**Authors:** Miguel A. Gonzalez-Lozano, Patricia Klemmer, Titia Gebuis, Chopie Hassan, Pim van Nierop, Ronald E. van Kesteren, August B. Smit, Ka Wan Li

**Affiliations:** 1 Department of Molecular and Cellular Neurobiology, Center for Neurogenomics & Cognitive Research, Neuroscience Campus Amsterdam, VU University, Amsterdam, The Netherlands; 2 Center for Proteomics and Metabolomics, Leiden University Medical Center, Leiden, The Netherlands

## Abstract

Development of the brain involves the formation and maturation of numerous synapses. This process requires prominent changes of the synaptic proteome and potentially involves thousands of different proteins at every synapse. To date the proteome analysis of synapse development has been studied sparsely. Here, we analyzed the cortical synaptic membrane proteome of juvenile postnatal days 9 (P9), P15, P21, P27, adolescent (P35) and different adult ages P70, P140 and P280 of C57Bl6/J mice. Using a quantitative proteomics workflow we quantified 1560 proteins of which 696 showed statistically significant differences over time. Synaptic proteins generally showed increased levels during maturation, whereas proteins involved in protein synthesis generally decreased in abundance. In several cases, proteins from a single functional molecular entity, e.g., subunits of the NMDA receptor, showed differences in their temporal regulation, which may reflect specific synaptic development features of connectivity, strength and plasticity. SNARE proteins, Snap 29/47 and Stx 7/8/12, showed higher expression in immature animals. Finally, we evaluated the function of Cxadr that showed high expression levels at P9 and a fast decline in expression during neuronal development. Knock down of the expression of Cxadr in cultured primary mouse neurons revealed a significant decrease in synapse density.

Chemical synaptic transmission constitutes the major mode of communication in the brain. Mature synapses are composed of a transmitter-releasing presynaptic element and a signal-receiving and -processing postsynaptic compartment^1^, that are estimated to contain over 2000 different proteins^2^. In mammals, synaptogenesis starts prenatally and proceeds well beyond birth, with rapid and specific changes of synapse numbers, synapse morphology and protein expression at early juvenile stage through adolescence^3^,^4^. In particular, synapse number in rodents rapidly increases in the first three weeks after birth^4^, and the typical synaptic structure with normal sized synaptic vesicles and postsynaptic thickening, can be observed at the end of the first postnatal week^5^. These developmental changes allow neurons to establish connections with appropriate partners, to prune the initial wiring of extensive synaptic connections into a more refined and restricted number of relatively stable synaptic contacts, and to tune the functional properties of synapses to prepare them for adult function and plasticity^6^,^7^.

Synaptogenesis also involves the timed expression and functional incorporation of proteins. Both excitatory and inhibitory synapse formation are initiated by contacting transsynaptic adhesion molecules, as exemplified by the presynaptic neurexin - postsynaptic neuroligin interaction^8^,^9^. This contact leads, in the case of excitatory synapse formation, to postsynaptic NMDA receptor recruitment to the nascent synapse^10^. Subsequent AMPA receptor recruitment makes the synapse functional and allows rapid synaptic transmission^11^. Finally, receptor subunit switching, for instance replacing NMDA receptor subunit Grin2b with Grin2a^12^,^13^, renders a physiological mature synapse^14^. It is generally accepted that in addition to these examples the synaptic proteome undergoes extensive developmental changes that underlie the progression of synaptogenesis and synapse maturation^15^. Conversely, dysregulated synaptic protein expression and subsequent disturbance of timely interactions of proteins during development have been linked to impaired synaptic function in several disorders, such as autism, schizophrenia and several forms of mental retardation^16^,^17^.

To establish insight into synaptic protein expression profiles that cover the diversity of the synaptic proteome during the entire span of development, a comprehensive proteomics approach is required. In the present study, an iTRAQ reagent-based proteomics workflow was employed for the relative quantitation of synaptic membrane proteins across 8 time points of juvenile, adolescent and adult mouse brain cortex development in a single 8-plex set of experiments. iTRAQ reagent-based proteomics is particular suitable for time series experiments due to the possibility of multiple sample labeling and analysis in one experiment. Quantification depends on the measurement of the iTRAQ signature ions generated from the tandem mass spectrometry (MS/MS) of the precursor ion. In such a complex sample, i.e., the synaptic membrane preparation of the mouse cortex, the number of quantified peptides and the accuracy of quantification critically depend on the capacity of the peptide separation system. We employed the OFFGEL isoelectric focusing system as first dimension separation^18^.

In total, 3 independent biological replicates were analyzed, identifying 1978 proteins, of which 1560 proteins were present in all three replicates and 696 showed statistical differences over time. These data provides a new insight for generating novel hypotheses of molecular processes underlying synaptogenesis and synapse maturation.

## Results

### Quantitative proteomics workflow

Using the 8-plex iTRAQ reagents, we performed a time-series relative quantification of the brain cortical synaptic membrane proteome of juvenile postnatal days 9 (P9), P15, P21 and P27, adolescent P35 and adult P70, P140 and P280 mice. As the postsynaptic density is crucial for the biochemical enrichment of synaptic membranes, the main target of this study, the juvenile age P9 was chosen as the earliest time point.

First, cortical synaptic membrane fractions were isolated, and peptides derived from trypsin protein digestion were fractionated by Strong Cation Exchange (SCX) chromatography, to remove the bulk of free iTRAQ label and other reagents that might interfere in subsequent analysis. These fractions were pooled, desalted and separated by OFFGEL electrophoresis, in which each OFFGEL fraction was analyzed by Liquid chromatography-Mass spectrometry (LC-MS).

The standard OFFGEL protocol (provided by Agilent Technologies) resulted in 70% of the distinct peptides found in single fractions (Experiment 1). Previous studies suggested that better separation can be achieved^18^. Therefore, the total focusing duration was increased to 80 kVh instead of the standard 50 kVh. Thereby separation was improved to 80% of distinct peptides contained in single fractions (Experiment 2 and 3).

In total, three independent biological samples of synaptic membranes per time point were analyzed using OFFGEL methodology. A total of 1978 proteins were identified with a high confidence protein score (≥unused ProtScore 3) from the combined search of all data in the three individual replicates (Supplementary sTable 1). The unused ProtScore is a sum of the scores from all peptides matched to the protein. As the maximum score that can be assigned to a single peptide is 2, the ProtScore of 3 was derived from ≥2 peptides. The complete list of proteins and peptides identified in the combined search of all three experiments together can be found in Supplementary sTable 2, and individual searches for experiment 1, 2 and 3 in Supplementary sTables 3, 4 and 5, respectively.

**Figure 1:**
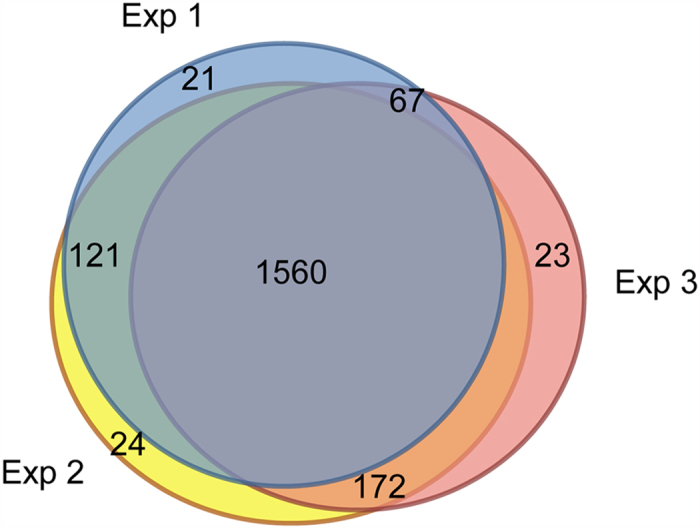
Venn diagram showing the distribution of the number of quantified proteins over the experiments. Represented are all 1978 proteins quantified with a high confidence protein score (ProteinPilot unused ProtScore ≥3, see main text) across all three experiments. In total 1560 proteins are common between the data sets.

For adequate statistical analysis, we only considered identified proteins with high confidence and present in all three replicates with no missing values at any time point. In total 1560 proteins were shared between the three OFFGEL experiments (Fig. 1).

### Unbiased interpretation of functional protein groups with significant temporal differences

**Figure 2:**
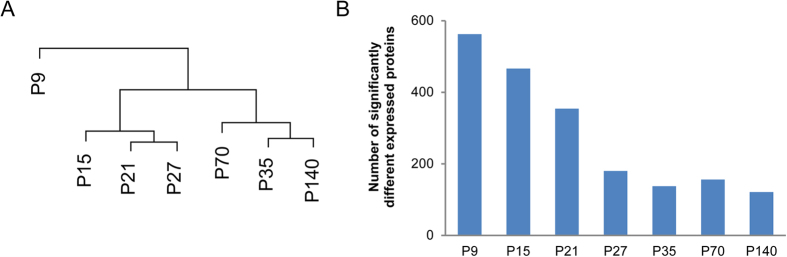
Protein expression relationship between samples of different developmental stages. (**A**) The average values of the protein levels of the different age groups were hierarchically clustered. The vertical distance connecting two samples within the dendrogram reflects similarity between the samples. (**B**) Number of proteins that were significantly different in abundance between P280 and other developmental stages (one-sample student’s t-test, p-≤0.05). P280 was taken as reference for the relative quantification of the other 7 age groups.

To reveal the developmental changes in protein expression, the protein abundances of the adult mouse sample at P280 were taken as reference to compare with and to visualize the levels of proteins of the other 7 age groups. Firstly, hierarchical clustering was performed to examine the relative similarities of different developmental stages. This analysis was performed on the mean expression levels (log2-transformed) of the three biological replicates. Samples taken from adolescent and adult animals clustered into separate groups (Fig. 2), and were both distant from animals of the youngest developmental stages (Fig. 2a). Secondly, whereas more than 350 proteins are differentially expressed between P280 and the juvenile stages (P9, P15 and P21), only around 150 differentially expressed proteins were observed between P280 and the adult stages (Fig. 2b). Thus, as expected, major changes in protein levels take place in juvenile stages and these differences in abundance get smaller towards the fully matured state, as was previously reported^19^.

**Figure 3:**
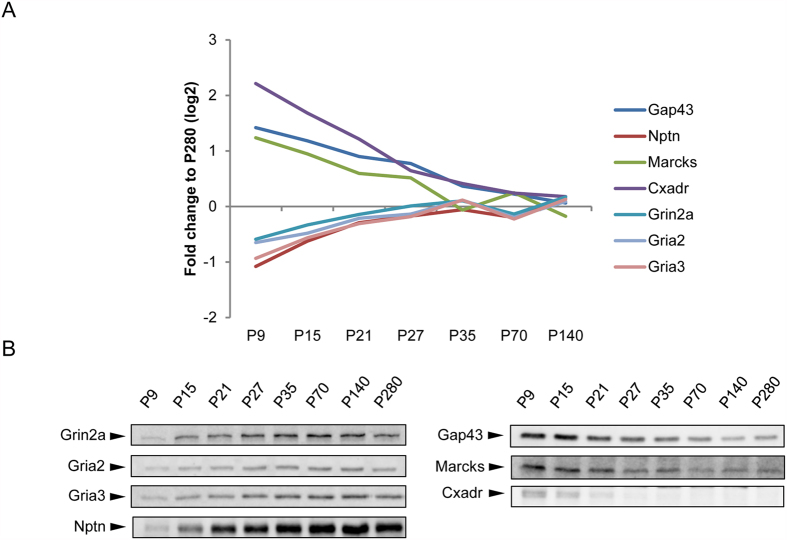
Relative iTRAQ levels and SDS-PAGE immunoblot analysis of selected synaptic proteins. (**A**) The abundance profile of each protein depicted is presented as the ratio of signal intensity (fold difference on log2 scale) of the mean of three biological independent iTRAQ sets compared to the reference sample (P280). (**B**) SDS-PAGE immunoblot analysis of the selected proteins with an increasing (left) or decreasing (right) expression pattern across time points.

To reveal proteins that showed significant change in abundance over time, Bayesian Estimation of Temporal Regulation (BETR) statistical testing was employed, which takes correcting for multiple testing into account that is obligatory with samples of large size^20^. This resulted in 696 proteins showing a significant increase or decrease in levels over time (BETR ≤ 0.001; Supplementary sTable 1). As example, proteins with significantly high fold differences at P9 versus P280 are listed in Table 1. Importantly, there is a strong overlap between the most differentially expressed proteins reported herein with previous studies^15^. For instance Fabp7, which showed the highest ratio between P9 and P280 animals, is a protein expressed in neural stem and progenitor cells, reaches a maximum on E14 and gradually decreases after birth^21^,^22^. Also, the strong decrease in expression of Dcakd was previously reported^15^, as holds for proteins having a role in neurite outgrowth (e.g., Marcks and Gap-43; Fig. 3A). To confirm the results obtained by iTRAQ based proteomics we performed immunoblotting analysis for several well known synaptic proteins with different patterns of expression, showing good agreement between both methods (Fig. 3B, Supplementary sFigure 1).

To capture the functional significance of protein regulation in an unbiased manner, we interrogated the data with Ingenuity Pathway Analysis (IPA). Data from P9 to P280, the time range that generally gives the largest fold differences, was compared for different categories: (1) disease and disorders, (2) molecular and cellular functions, and (3) physiological system development and function (Supplementary sTable 6A). The low p-values for neurological and psychological disorders, nervous system development, and function and behavior, reflect the nature of the synapse preparation^23^. To probe the alteration of underlying biological processes, we interrogated specifically the diseases and function annotation in IPA. The top 5 for functional annotation and for diseases and disorders annotations are shown in Table 2. All the information generated from the IPA analysis, including protein identities for each process, are shown in supplementary sTable 6B. As expected, the main biological processes differentially regulated during neuronal development were neurotransmission and neuroplasticity (LTP), the latter may underlie the synaptic features of learning and cognition, not yet fully developed in immature synapses. Interestingly, abnormal processes of movement disorders and seizure apparently share similarity to the proteome profile of immature synapses.

**Figure 4:**
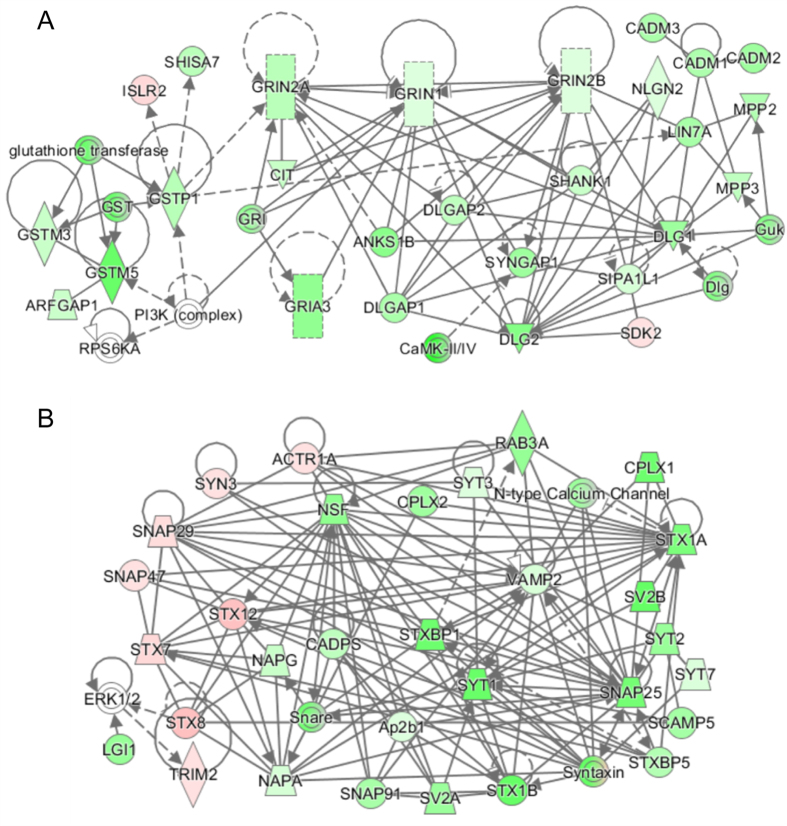
Unbiased construction of protein sub-networks by IPA from proteins showing significant developmental changes. (**A**,**B**) show mainly the networks of post- and pre-synaptic proteins, respectively. Green color intensity indicates the level of down-regulation and red for up-regulation in P9/P280 comparison. Continuous and discontinuous connections indicate direct or indirect interaction, respectively.

Biological processes are often driven by specific multi-protein machineries. It is expected that proteins contained in such interaction are co-regulated. Examples are for instance the ribosome, of which the residing proteins are all co-regulated during development in this study (data not shown). In addition, synaptic proteins may assemble into larger networks, in which residing proteins together may regulate function. To reveal how protein networks are altered during development, we examined the regulation of the main pre- and postsynaptic networks as predicted by IPA. The biological process neurotransmission is found to be the main process with proteins still at low levels at P9 (Table 2). In accordance, a lower level of the canonical NMDA receptor protein complex and other postsynaptic proteins are revealed at P9 (Fig. 4A), reflecting a feature of the immature nature of the synaptic preparation. Members of GST known to be involved in detoxification process were also down regulated, and this pathway is indicated as having an indirect interaction with the postsynaptic proteins. Similar to these postsynaptic proteins, core presynaptic proteins at P9 were also present at lower levels. For instance, low levels of proteins that control or regulate membrane fusion and membrane retrieval, involving the t- and v-SNARE proteins and the proteins of endocytosis can be observed (Fig. 4B). Interestingly several non-canonical SNARE proteins, namely Snap 29/47, Stx 7/8/12 and Syn3, were oppositely regulated at P9 with higher expression levels. Syn3 has been reported to show a high level of expression in growth cones of immature neurons^24^, however, the functions and localization of the non-canonical SNARE proteins are less clear. IPA reveals an extensive connection of Snap29 to other canonical presynaptic proteins. It was reported to have broad tissue expression and appears to participate in many fusion events including Golgi, endosome and lysosome membranes in many cell types. It is suggested as a negative regulator of SNARE complex disassembly after fusion^25^,^26^. Snap47 on the other hand seems to have function similar to Snap25 (i.e., having a role in synaptic vesicle fusion), and in addition plays role in axon branching^27^,^28^. The roles of various Stx isoforms in neurotransmission have been poorly documented, but are believed to have functions specific to synaptic vesicle fusion and recycling^29^.

**Figure 5:**
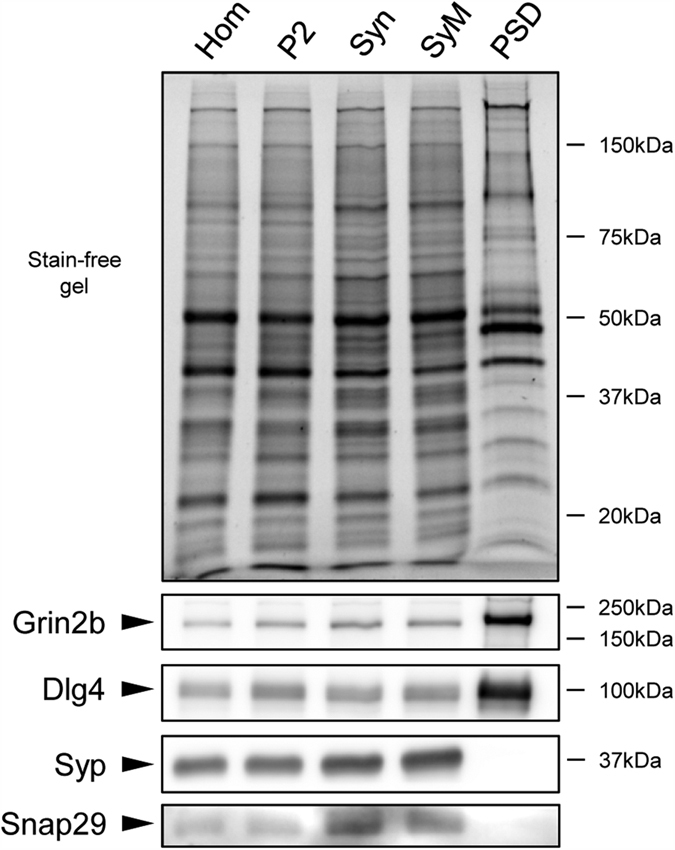
SDS-PAGE immunoblot and stain-free gel images of different biochemical fractions of adult mouse cortex. Samples of different enriched subfractions were resolved on SDS-PAGE, and then immunoblotted for specific synaptic markers. NMDA receptor 2b (Grin2b) and Dlg4 proteins are markers of postsynaptic density fraction; synaptophysin (Syp) is a marker of the presynaptic terminal. Hom: homogenate; P2: pellet 2; Syn: synaptosome; Sym, synaptic membrane; PSD: Triton X-100 insoluble postsynaptic density fraction. Normalization of the protein input was performed using the stain-free gel.

The connection of Snap29 to other canonical presynaptic exocytosis proteins suggests that it is a presynaptic protein as well. In order to test this possibility, we compared the relative abundance of Snap29 to several canonical synaptic proteins across biochemical fractionated synaptic organelles as an initial characterization of their spatial distribution in mature synapses (Fig. 5). Presynaptic (Syp) and postsynaptic (Dlg4 and Grin2b) proteins showed their typical enrichment in synaptosome and PSD fraction^30^,^31^,^32^,^33^, respectively. Snap29 spatial distribution generally followed the same pattern as Syp, in accordance to the vesicle/SNARE-related functions mentioned above. This distribution pattern supports the notion that, in mature neurons, Snap29 is a presynaptic protein. The functional role of these preferentially early expressed SNARE proteins remains to be determined.

### Presynaptic proteins

**Figure 6:**
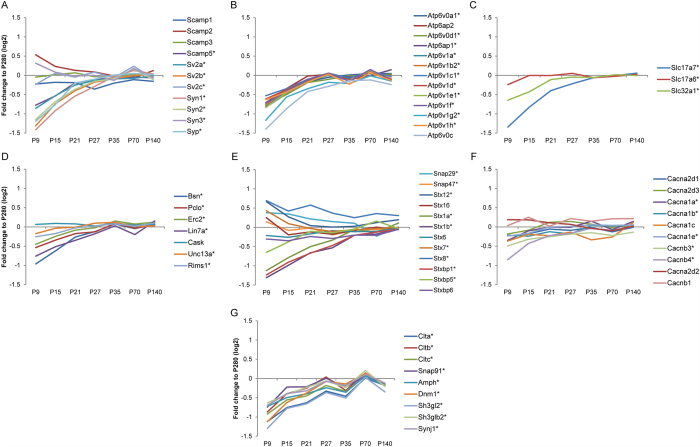
Relative levels of typical presynaptic proteins. The abundance profile of each protein depicted is presented as the ratio of signal intensity (fold difference on log2 scale) of the mean of three biological independent iTRAQ sets compared to the reference sample (P280). Synaptic proteins were assigned to specific functional groups, such as synaptic vesicle proteins (**A**), proteins important for docking (**D**), proteins involved in priming and exocytosis (**E**), and proteins implicated in clathrin-mediated endocytosis and recycling (**G**). In addition, the expression profile of the different subunits of the vacuolar ATPase is shown (**B**), vesicular transporters (**C**) and calcium channels (**F**). The asterisks at the protein names indicate significant difference in level over at least two time points (BETR ≤ 0.001; Supplemental Table 1).

In addition to IPA analysis, several major functional classes of presynaptic proteins members were identified (Fig. 6). Interestingly, synaptic vesicle proteins showed increasing levels with slight differences in the order of extent of regulation (Fig. 6A), with some exceptions, namely synapsin 3 (Syn3), as previously described^24^, and Scamp2. All vesicle-type proton ATPase subunits showed an increase in level throughout brain development with only small variation between the subunits (Fig. 6B). The tight co-expression of the subunits reflects the fact that together these proteins form a functional proton-pump in the synaptic vesicle membrane^34^. Interestingly, glutamate and GABA vesicular transporters (Fig. 6C) show different expression from P9 to P27, to converge in adolescence (P35), the critical period of regulation of the excitatory-inhibitory balance^35^,^36^. Bassoon (Bsn), piccolo (Pclo) and Erc2 form a complex that binds synaptic vesicles and plays a role in their transport to the docking site^37^, and also showed a similar increase in levels (Fig. 6D). Cask showed no change, whereas Lin7A and Munc13 (Unc13a) were increased. On the other hand, levels of proteins involved in priming and exocytosis, e.g., syntaxin 1 (Stx1) and Stxbp1 were more variable (Fig. 6E). Snap 29/47 and Stx 7/8/12 follow a decrease in expression levels along all time point. Voltage-dependent calcium channel subunits (e.g. Cacnb3 and Cacnb4) followed same patterns of increasing expression or did not change (e.g. Cacnb1 and Cacna2d1), except for some subunits, such Cacna1c, that shows a diminished level around P35 (Fig. 6F). Proteins important for endocytosis and recycling of synaptic vesicles^38^,^39^, such as clathrins (Clta, Cltb, Cltc), dynamin 1 (Dnm1) and amphiphysin 1 (Amph), increase in levels over time in a similar fashion (Fig. 6G). Taken together, proteins of the presynaptic machinery show an overall increase in levels during development, reflecting the increase in numbers and/or maturation of the presynaptic terminal and the onset of synaptic vesicle release cycle. Increase in numbers of synaptic vesicles during development, has been shown previously^5^,^40^, and might explain the observed increase of synaptic vesicle proteins that is shown here.

### Postsynaptic proteins

**Figure 7:**
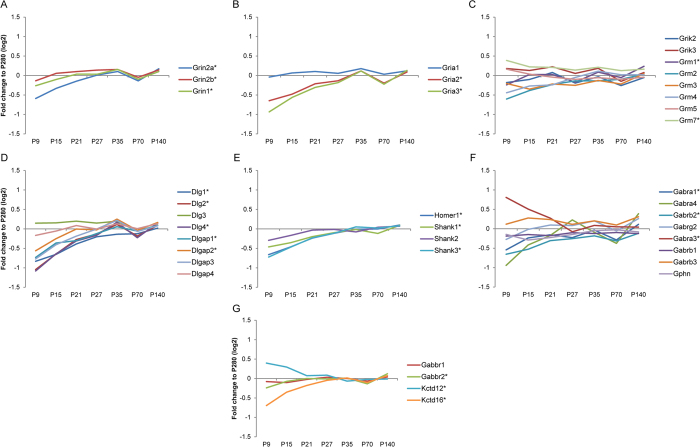
Relative levels of typical postsynaptic proteins. The abundance profile of each protein depicted is presented as the ratio of signal intensity (fold difference on log2 scale) of the mean of three biological independent iTRAQ sets compared to the reference sample (P280). Synaptic proteins were assigned to specific functional groups, such as ionotropic glutamate receptors; NMDA- (**A**), AMPA- (**B**), kainate-type and metabotropic glutamate receptors (**C**), scaffolding proteins of the PSD (**D,E**), inhibitory GABA_B_ and GABA_A_ receptor subunits and gephyrin (**F,G**). The asterisks indicate proteins significantly regulated over at least two time points (BETR ≤ 0.001; Supplemental Table 1).

The postsynaptic element of a glutamatergic synapse contains a densely packed protein structure containing various types of glutamate receptors, scaffolding proteins, signaling proteins, adhesion molecules and cytoskeletal proteins^32^. The tetrameric ionotropic AMPA- and NMDA-type glutamate receptors in most cases are comprised of two different subunit types^41^. The NMDA receptor is composed of subunit GluN1 (Grin1) and either the GluN2A (Grin2a) or GluN2B (Grin2b) subunit. Grin1 and Grin2b abundance levels increase slightly over time, whereas Grin2a has clearly a lower expression level in young animals, which increases over time (Fig. 7A), suggesting that the NMDA receptor subunit composition shifts from predominating Grin1-Grin2b in young animals, to Grin1-Grin2a in adult animals, with a described corresponding change in NMDA receptor channel properties^13^. For AMPA receptors, the subunit GluA1 (Gria1) has constant expression pattern, whereas GluA2 (Gria2) and 3 (Gria3) have a lower expression level at early developmental stages, which increases over time (Fig. 7B). Other glutamate receptors, such as the kainate-type glutamate receptor and the metabotropic glutamate receptors, mostly showed constant levels throughout all stages (Fig. 7C). Important contributors to the architecture of the postsynapse are scaffolding proteins, including members of the PSD-95 family (Dlg), together with associated proteins (SAPAPs or Dlgap) and SH3 domain and ankyrin repeat proteins (Shanks). These are involved in clustering of glutamate receptors and cell adhesion molecules, as well as the recruitment of signaling molecules and the anchoring of these to the cytoskeleton^42^. Also these proteins showed differential developmental expression patterns (Fig. 7D,E) and generally increased in levels over time. There is a remarkable similarity in the general expression pattern of AMPA, NMDA receptor subunits and family members of PSD-95 (such as Dlg1, 2, 4 and all Dlgap), all following a gradual increment with a maximum at P35, and then leveling off at P70. This event seems to be correlated with the maturation and synapse formation that occurs until adolescence, and the synaptic refinement that takes place until that particular period^36^.

The inhibitory metabotropic GABA_B_ receptors, present in excitatory synapses, and ionotropic GABA_A_ receptors, in inhibitory synapses^43^, showed variable expression profiles throughout the developmental stages (Fig. 7F,G). The increase in expression levels of GABA_A_ receptor α1 (Gabra1), β2 (Gabrb2) and γ2 (Gabrg2) subunits, whereas others such as α3 decrease (Gabra3), indicates a subunit composition shift towards the major adult isoform (α1, β2 and γ2)^44^. Gephyrin (Gphn), which is considered a major scaffolding protein at inhibitory synapses, did not show a significant regulation. In addition, the extrasynaptic GABA_A_ receptor α4 subunit (Gabra4) level was higher when neuronal maturation neared completion, as has been described previously^45^.

GABA_B_ receptors remained unchanged, or were increased slightly over time, such as in case of the GABA_B_ receptor 2 (Gabbr2, Fig. 7G). Moreover, potassium channel tetramerization domain (KCTD) 12 and 16, part of GABA_B_ receptor complex, were found decreasing and increasing in levels, respectively, until stabilization around P35 in accordance with the high expression of KCTD12 in fetal brain^46^.

### Adhesion molecules

**Figure 8:**
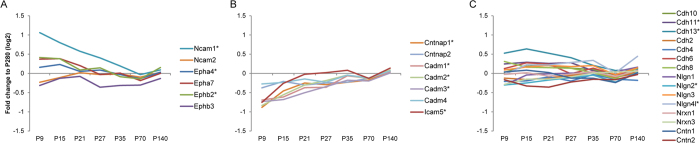
Relative levels of cell adhesion molecules. The abundance profile of each protein depicted is presented as the ratio of signal intensity (fold change on log2 scale) of the mean of three biological independent iTRAQ sets compared to the reference sample (P280). Major classes of cell adhesion molecules were grouped based on their expression patterns into (**A**) overall decreasing levels (ephrin receptors, NCAM), (**B**) increase in levels (SynCAM, Contactin, ICAM), or (**C**) not changed (cadherins, neuroligins, neurexins and contactins). The asterisks indicate proteins significantly regulated over at least two time points (BETR ≤ 0.001; Supplemental Table 1).

The pre- and postsynaptic elements are held together by interactions of cell adhesion molecules present in the opposite synaptic membranes. During synaptogenesis, these proteins are involved in target recognition, induction of pre- and post-synaptic differentiation, and in the alignment of pre- and postsynaptic neurotransmitter release and reception machineries^8^. The best-characterized cell adhesion molecules include neuroligins-neurexins, ephrins-ephrin receptors, the Ig superfamily, cadherins, and the neurofascin family. The expression patterns of adhesion molecules (Fig. 8) reveal high diversity in the immature synapses at P9 even between members of the same family. Whereas, most proteins belonging to the cadherin superfamily maintained a fairly constant level over the whole developmental period, Ig superfamily members show highly diverse protein expression patterns. For example, Ncam1 was found highly abundant at P9 and decreased towards maturation, whereas Ncam2, SynCAMs (Cadm) and Icam5 were slightly increased in expression (Fig. 8A,B).

### Functional validation of a highly regulated protein: Cxadr

**Figure 9:**
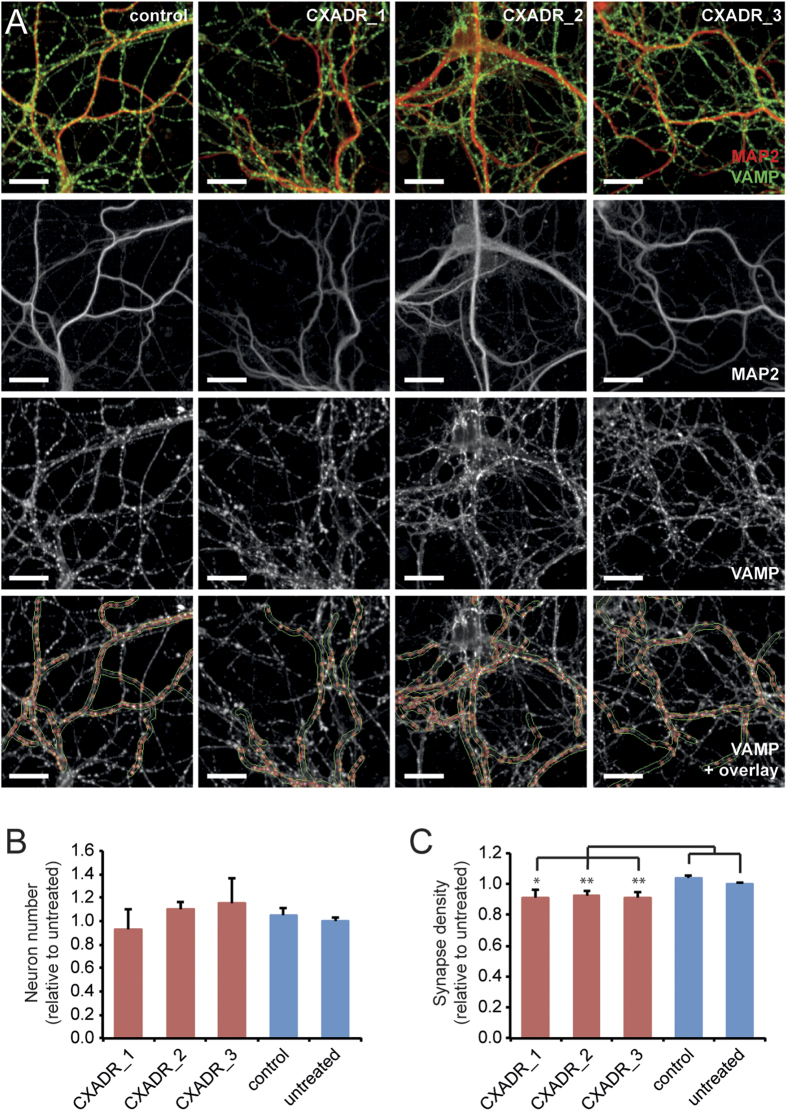
Cxadr knockdown affects synapse formation *in vitro*. (**A**) Images of primary mouse hippocampal neurons transduced with a negative control shRNA or with one of three shRNAs against Cxadr. Top panels show image selections with MAP2 staining (red) to identify dendrites and VAMP staining (green) to identify presynaptic spots. The MAP2 channel (row 2) was used to create a dendritic mask, and the VAMP channel (row 3) was used to identify synaptic spots within the dendritic mask region. Examples of the dendritic masks and identified synaptic spots are shown in row 4 on top of the VAMP staining. Scale bars: 10 μm. (**B**) Transduction with shRNAs did not affect neuron numbers. (**C**) All three shRNAs against Cxadr reduced synapse densities. Cxadr shRNAs, n = 5; control shRNA, n = 8; untreated cells, n = 15. *p < 0.05; **p < 0.01.

Among all quantified proteins, Coxsackie virus and adenovirus receptor (Cxadr) showed one of the highest ratios between the P9 and P280 animals (Table 1). Cxadr acts as a cell adhesion molecule interacting through its PDZ-domain binding motif with intracellular scaffold proteins including PSD-95, and it has been implicated in the regulation of neurite extension^47^. The relatively high expression levels of Cxadr at P9 and its fast decline in expression soon thereafter suggest that it may play a role in synapse formation or maturation. To test this, we knocked down the expression of Cxadr in cultured primary mouse neurons. Neurons were obtained from E18 mouse embryos and transduced at DIV2 with lentiviruses expressing either one of three different shRNAs against Cxadr or a scrambled negative control shRNA. At DIV14, cells were fixed, stained for dendritic and synaptic markers, and analyzed using high-content microscopy. Neuron numbers, dendrite length and synapse numbers were quantified unsupervised and fully automated using Columbus software (PerkinElmer) (Fig. 9A). None of the shRNAs significantly reduced neuron numbers, indicating normal cell viability (Fig. 9B). All three shRNAs against Cxadr however significantly reduced synapse densities, i.e., the number of synapses per dendrite length, compared with either control shRNA-transduced cells or with untreated cells (Fig. 9C). This reduction was on average 8.4% ± 4% (shRNA #1, p < 0.05; shRNAs #2 and #3, p < 0.01).

## Discussion

To investigate the molecular development of the synapse, we have used OFFGEL separation of proteins and an iTRAQ-based mass spectrometry analysis of the synaptic membrane fraction of the mouse cerebral cortex. iTRAQ technology enabled comparing eight different developmental stages in a proteome-wide manner in a single experiment. Although synaptic proteins have been studied in depth during critical periods of development^15^,^48^, the adaptation of the synaptic proteome throughout various stages of postnatal development has not been described. This is the first study of the relative quantification of synaptic proteins at eight distinct time points of postnatal brain development, including juvenile, adolescent and adult stages.

This study was performed with three independent biological replicates per developmental stage divided over three iTRAQ experiments. Among 1978 identified proteins, 1560 were common to the three iTRAQ experiments, and 696 showed statistically significant change in abundance in two or more developmental stages. The analysis with IPA reveals that neurotransmission and tissue development are the main regulated processes during neuronal maturation. A higher number of regulated proteins is observed in juvenile and adolescent animals (P9 - P27), which stabilized in adults (P70, P140, P280), which seems to be a general pattern observed in different studies^19^,^40^. In the still developing brain (at P9 or P15) the machinery for protein synthesis was more abundant, consistent with the notion that a development puts a higher demand on the supply of new (synaptic) proteins^49^,^50^.

The majority of presynaptic proteins showed gradual increase in levels, which is in line with previous studies^40^,^48^. The synaptic vesicle integral membrane proteins such as V-ATPases, SV2, Syn and Syp have a low expression levels at P9, and together these increased to a constant level at P27. This was accompanied with similar changes in levels of bassoon (Bsn), piccolo (Pclo) and Erc2, organizers the active zone. Proteins involved in endocytosis, such as clathrin (Clt), dynamin (Dnm1) and synaptojanin (Synj1), also increased and stayed at a constant level at around P27. As immature synapses at P9 grow in size, they become filled with larger numbers of synaptic vesicles and generate a wider active zone present at P27^5^,^40^. Accordingly, many proteins of the vesicle release machinery, e.g., synapsin (Syn) and synaptophysin (Syp), increased in abundance during development. Proteins involved in docking of synaptic vesicles to the active zone, however, did not show such a clear pattern of abundance change. For instance, Cask showed constant levels throughout all time points, whereas Lin7A and Stxbp1 abundance increased from P9 onwards. On the other hand, the non-canonical SNARE protein Snap29, with a presynaptic enrichment, has a higher expression level at P9 that decreases over time. Preferential early expression patterns were also observed for Snap47 and Stx 7,8,12. The functional significance of differential expression patterns in developmental changes of distinct vesicle release machinery proteins is of interest and remains to be investigated.

Developmental changes of pre- and postsynaptic elements likely show coordinated expression to match full synapse maturation. Indeed, the typical postsynaptic scaffolding proteins SAP-97 (Dlg1), PSD-93 (Dlg2) and PSD-95 (Dlg4) showed expression patterns that are similar to those of the presynaptic synaptic vesicle proteins, synaptic vesicle glycoprotein and secretory carrier-associated membrane protein. SAP-102 (Dlg3) showed a different expression pattern as described previously^51^ with high expression in young animals (P9) and lower levels in adult animals (P280). This protein mediates synaptic trafficking of AMPA- and NMDA-type receptors during synaptogenesis^52^, a process that is also linked to the developmental shifts of NMDA and AMPA receptor subunits. In later stages, PSD-95 takes over the functions of SAP-102 (Dlg3), mediating the developmental increase of AMPA receptor transmission and the replacement of Grin2b with Grin2a-containing NMDARs^53^,^54^.

Co-regulation or distinct expression patterns of proteins over time might give clues to shared roles either during synapse formation, maturation or in synaptic plasticity. Proteins with highest P9 to P280 ratio are listed in Table 1. Signaling proteins with known involvement in neurite extension and axon growth of cortical neurons, such as the Calcium-calmodulin binding PKC substrate proteins Marcksl1 and Gap-43^55^,^56^, were relatively abundant early in development. In the adult brain, these proteins regulate synapse morphology through remodeling of the actin cytoskeleton. Levels of several isoforms of the microtubule-associated protein family strongly decreased over time (e.g. Map1b), whereas other specific tubulin-cytoskeleton associated proteins (e.g. Tppp) increased as previously demonstrated in the visual cortex during adolescence^48^. These findings suggest discrete functions of Tubulin-associated proteins during brain development, which needs further investigation.

In addition to the proteins previously reported involved in neurite outgrowth and synaptogenesis, there are a number of proteins currently not known to be associated with synapse formation and/or maturation. One of the most regulated proteins identified was the cell adhesion molecule Coxsackie virus and adenovirus receptor (Cxadr). The low expression and restricted distribution of Cxadr in mature brain indicates that this protein might play a prominent role in immature synapses. In general, the functionality of cell adhesion molecules is diverse; they stabilize synaptic contacts, promote neurite outgrowth and act in path-finding, control of synaptic formation and are involved in regulation of synaptic transmission and plasticity. In order to test the functional implication of Cxadr in synapse development, we knocked down the expression of Cxadr in cultured primary mouse neurons. All three shRNAs against Cxadr significantly reduced synapse densities, suggesting that Cxadr play a role in synapse formation or maturation, which goes in parallel with the expression profile of the protein.

Fatty acids are critical structural components of the brain and essential for normal brain development, including the generation of neuronal membranes, differentiation and migration^57^, and axon outgrowth mechanism are controlled by phospholipid-mediated signaling^58^. In line with this, we found Lipid phosphate phosphatase-related protein type 3 (Lppr3), Low-density lipoprotein receptor-related protein 1 (Lrp1) and Fatty acid synthase (Fasn), all involved in lipid homeostasis, highly expressed in juvenile synapses at P9. Remarkably, Fatty acid-binding protein 7 (Fbp7) has the largest ratio between P9 and P280 animals. Specifically localized in neural cells, this protein has been described to play an essential role in neurogenesis^21^, and has been associated with psychiatric illnesses, such as schizophrenia and autism^22^,^59^.

In particular during adolescence, important changes in cortical architecture and function occur, such as regulation of the neuronal excitatory-inhibitory balance^36^,^60^. Our data enable to follow specific protein expression changes that are taking place during this period. For instance, different expression of vesicle transporters (e.g. vesicular glutamate transporter 1 (Slc17a7) and 2 (Slc17a6)) and regulation of excitatory and inhibitory receptors (e.g. GluA2/3 of AMPA receptor, KCTD12/16) occurs at P35 (adolescence). Adolescence is generally regarded as a vulnerable period in the development of mental illnesses, such as anxiety, mood disorders or schizophrenia^60^. Expression profiles of disease-associated proteins in this critical period may help to better understand that synaptic molecular basis of disease development. Of interest is Cacna1c (Cav1.2 alpha subunit 1c), which is associated with schizophrenia^61^. This protein shows an increasing abundance during early development, but in contrast with the other calcium channels subunits, has a lower expression at P35, after which it is then stabilized. The functional implication of this particular regulation is still unknown and might be considered for further analysis. Conversely, proteins with similar critical period patterns may be considered as potential risk proteins and studied further in that context.

Finally, it has to be considered that this study does not distinguish between the large diversity of neuronal cell types, including the vast majority of excitatory neurons^62^ and the different types inhibitory cells. These different cortical cell types possess particular developmental properties. The results shown here represent the average protein changes occurring in all cortical synapses during development. Consequently, neuron type-specific expression or developmental regulation of proteins in sparse neuron types might become too diluted to be detected.

In conclusion, proteome analysis of synapses in the developing brain as described here might aid in understanding the underlying molecular mechanisms of synapse formation and maturation. Developmental changes in protein levels are likely indicative for a functional role at a specific stage in synaptogenesis. The synaptic membrane fraction used here consists in principle of pre- and postsynaptic plasma membrane proteins and proteins that adhere to those. The development of a specific synapse ontology would be instrumental in generating hypotheses on the roles of distinct synaptic proteins. Besides this, various proteins discussed may be likely candidates for further functional and/or synaptic developmental studies.

## Materials and Methods

### Animals

C57Bl/6J mice were bred in our facility. Animals were decapitated on postnatal days P9, P15, P21, P27, P35, P70, P140 or P280. Following decapitation, the brains were removed and the cortex was dissected. The tissue was rapidly frozen and stored at −80 °C until further use. These experiments were approved by the animal ethics committee of the VU University. The methods were carried out in accordance with the approved guidelines.

### Sample Preparation

Synaptic membranes were isolated from a single mouse cortex as described previously^30^,^31^, except for mice of postnatal day 9, of which cortices of two animals were pooled to obtain sufficient tissue for subsequent analysis. In short, brain tissue was homogenized in ice-cold 0.32 M sucrose and centrifuged at 1,000 × g for 10 min. The supernatant was loaded on top of a sucrose gradient consisting of 0.85 M and 1.2 M sucrose. After ultracentrifugation at 100,000 × g for 2 h, the synaptosome fraction was collected at the interface of 0.85/1.2 M sucrose and then lysed in hypotonic solution. The resulting synaptic membrane fraction was recovered by ultracentrifugation using the sucrose step gradient as stated above. Synaptic membranes were harvested at the 0.85/1.2 M interface. Protein concentration was determined by a Bradford-based assay (Bio-Rad Protein Assay). 75 μg protein of synaptic membrane sample was dried in a SpeedVac and used for quantitative proteomics.

### Quantitative Proteomics

#### iTRAQ labeling

A single set of iTRAQ experiments contained 8 samples corresponding to the cortical synaptic membranes prepared from mice of different ages at postnatal days, P9, P15, P21, P27, P35, P70, P140 and P280. In total 3 sets of iTRAQ experiments were carried out on biologically independent samples. The previously described protocol for iTRAQ labeling of peptides was followed^63^. The dried synaptic membranes were resuspended in 28 μl of 0.5 M Triethylammonium bicarbonate buffer pH 8.5, containing 0.85% RapiGest (Waters associates). A 2 μl cleavage reagent (50 mM Tris[2-carboxyethyl] phosphine) was added and incubated at 55 °C for 1 h. A 1 μl Cys blocking reagent (50 mM S-methyl methanethiosulfonate) was added and incubated at room temperature for 10 min. Next, 5 μg of trypsin (Promega) dissolved in water was added and incubated overnight at 37 °C. Trypsinized peptides were tagged with one unit iTRAQ reagent (Sciex) dissolved in 80 μl isopropanol. After incubation at room temperature for 2 h the 8 samples were pooled and acidified with 5% TFA to pH 2.5 - 3.0. After 45 min, the sample was centrifuged and the supernatant was dried in a SpeedVac overnight.

#### Strong Cation Exchange (SCX) liquid chromatography

The dried iTRAQ sample was dissolved in loading buffer (20% acetonitrile, 10 mM KH_2_PO_4_, pH 2.9) and injected into a SCX column (polysulfoethyl A column from PolyLC). Peptides were eluted with a linear gradient of 0–500 mM KCl in 20% acetonitrile, 10 mM KH_2_PO_4_, pH 2.9, over 25 min at a flow rate of 200 μl/min. Fractions were collected at 1 min intervals. SCX fractions were pooled, desalted by solid phase extraction, dried, redissolved in water and subjected to OFFGEL separation.

#### OFFGEL peptide separation

Peptides were fractionated according to their pI using the Agilent OFFGEL 3100 fractionator (Agilent Technologies). Urea 1M was added to the IEF buffer instead of 5% glycerol mentioned in the manual. Commercially available IPG dry strips 24 cm, with a linear pH gradient ranging from 3–10 (GE-Healthcare) were used. The strips were rehydrated with 40 μl/well rehydration solution in the assembled device for 30 min. 150 μl of the pooled SCX fractions redissolved in water were loaded on each well. The cover fluid (mineral oil, Agilent Technologies) was added to both ends of the gel strip. The focusing method OG24PE01, as supplied by the manufacturer was used in the first OFFGEL experiment (Experiment 1). Typical voltage ranging from 500 to 4500 V was applied until 50 kVh was reached. The maximum current was set to 50 mA and the maximum power to 200 mW. In the subsequent two biological independent samples a longer focusing was applied till 80 kVh (Experiments 2 and 3). The higher volt-hour improved separation. In total 24 fractions were collected per OFFGEL experiment. Each OFFGEL fraction was pre-cleaned using the HLB μElution plates (Waters). The eluates were dried in a SpeedVac, redissolved in 20 μl 0.1% TFA, and subjected to LC-MS/MS analysis.

#### Reverse Phase liquid chromatography

Peptides were delivered with a FAMOS autosampler at 30 μl/min to a C18 trap column (1 mm × 300 μM i.d. column) and separated on a nano-C18 column (150 mm × 100 μm i.d. column) at 400 nl/min using the LC-Packing Ultimate system. Peptides were separated using linearly increasing concentration of acetonitrile from 5–40% in 50 min and to 90% in 1 min. The eluent was mixed with matrix (7 mg α-cyano-hydroxycinnaminic acid in 1 ml 50% acetonitrile, 0.1% TFA, 10 mM dicitrate ammonium) delivered at a flow rate of 1.5 μl/min and deposited off-line to the Applied Biosystems metal target every 15 s for a total of 192 spots using the probot (Dionex).

#### Mass spectrometry (MS/MS)

Samples were analyzed on a 4800 or 5800 proteomics analyzer (AB-Sciex). Collision-induced dissociation of peptides was performed at 1 kV with air as collision gas. Peptides with signal to noise ratio above 50 at the MS mode were selected for MS/MS, at a maximum of 20 MS/MS per spot. The precursor mass window was set at 200 relative resolution (FWHM). The MS/MS spectrum for each peptide was averaged from 2000 laser shots. The MS run time per OFFGEL fraction of 192 spots were about half to two days for 5800 and 4800 proteomics analyzer, respectively. Thus, the total 5800 proteomics analyzer measurement time for a single OFFGEL experiment was about 12 days. All the original machine generated data was deposited in PRIDES for public access (PXD004129).

#### Protein identification and quantification

Data obtained by mass spectrometry for each experiment and all three combined were analyzed with ProteinPilot software (version 4.5; Applied Biosystems; MDS Sciex) using the Paragon algorithm (version 4.5.0.0^64^) as the search engine. MS/MS spectra were searched against a mouse database without isoforms (Uniprot-Swissprot_2014/03). The parameters were set to iTRAQ 8-plex (peptide labeled), cysteine modification by MMTS, and trypsin digest. The detected protein threshold was set to 0.05 (10% confidence), bias correction was executed, and iTRAQ isotope correction factors were included. The fold change between labeled peptides (denominator: iTRAQ label 121, age P280) was calculated during the search (Supplementary sTables 2–5). The total number of proteins and peptides were obtained by performing a combined search with ProteinPilot including all three replicates (Supplementary sTable 2).

The resulting data for each biological replicate were combined, and reverse sequences and proteins with no quantitative values were removed. Furthermore, only proteins with a ProteinPilot unused value larger than 3 (peptide contribution values not claimed by another protein) across all three experiments were considered for posterior analysis (Supplementary sTable 1).

### Clustering and statistical analysis of protein expression profiles

Hierarchical clustering was used to visualize the similarity between the developmental stages. Time point clustering using Euclidean distance was performed on the log2-transformed means of the three replicates. To determine the number of statistically significant changed proteins between the reference (P280) and the other time points, a one-sample student’s t-test was executed in Perseus (MaxQuant, v1.5.2.6). The Bayesian Estimation of Temporal Regulation (BETR), which was initially developed for microarray data analysis^20^, was used to reveal statistical significant differences of the proteins in 3 replicates across the 8 developmental stages. Proteins were considered as significantly changed with a BETR ≤ 0.001. Finally, proteins with significant differences between P9 and P280 were imported into Ingenuity Pathway Analysis (IPA) for core analysis without user intervention. IPA identifies genes associated with a list of relevant functions and diseases, and the list is ranked according to the significance of the biological functions. Direct and indirect relationships were considered for the analysis from all data sources, mutation and species available with experimentally observed confidence level. The interaction networks were generated with a maximum of 35 molecules per network.

### SDS-PAGE Immunoblot analysis

Synaptic membrane fractions from individual animals were mixed with 5x SDS sample buffer and heated to 98 °C for 5 min. Proteins were separated on a 8% SDS-polyacyrlamide gel containing 0.5% TCE in a Mini-Protean Electrophoresis System (Bio-Rad). After electrophoresis, proteins in the gel were modified and scanned in the Gel Doc EZ imager (Bio-Rad)^65^. Proteins were electroblotted onto a PVDF membrane overnight at 40 V, and scanned in the Gel Doc EZ imager. Membranes were blocked with LI-COR blocking solution, incubated with primary antibodies at 4 °C overnight, and then incubated with a matched secondary antibody conjugated to either IRDye 680 (goat-anti-mouse) or IRDye 800 (goat-anti-rabbit) for 1 h. After washing, the blot was scanned with the Odyssey Fc imaging system (LI-COR Bioscience) and analyzed with Image Studio software (version 2.0.38). Differences in loading amount were corrected to (TCE-activated) total protein on the gel. In addition, samples from different subcellular compartments of adult mouse cortex were employed for immunoblot with secondary antibodies conjugated to Horseadish peroxidase and scanned with Femto ECL Substrate (Thermo Fisher Scientific).

The following antibodies were used for immunoblot analysis; Grin2a (1:1000, Abcam, ab14596), Grin2b (1:1000, Neuromab, 75-101), Gria2 (1:1000, Neuromab, 75-002), Gria3 (1:1000, Abcam, ab87609), Gap43 (1:1000, Sigma, G9264), Marcks (1:250, GenScript, A00511), Nptn (1:500, Gift from K. H. Smalla), Cxadr (1:500, obtained from GenScript, raised against the synthetic peptide PDLKGRVHFTSNDVC), PSD95 (Dlg4, 1:10000, Neuromab, 75-028), Syp (1:2000, GeneScript, A01307) and Snap29 (1:1000, Synaptic Systems, 111303).

### Primary neuron culture, Cxadr knockdown and high-content microscopy

Hippocampi were dissected from E18 wildtype C57Bl/6 mouse embryos. Neurons were plated in 96-well glass bottom plates (Cellstar, Greiner Bio-One, Frickenhausen, Germany) that were coated with poly-d-lysine (Sigma-Aldrich) and 5% heat-inactivated horse serum (Invitrogen). Cells were plated at a seeding density of 15k/well and cultured in Neurobasal medium supplemented with 2% B-27, 1.8% HEPES, 1% glutamax and 1% Pen Strep (all from Invitrogen) at 37 °C/5% CO_2_ for 14 days. Two days after plating, cells were transduced with lentiviral particles encoding one of three different shRNAs against Cxadr (Sigma; TRCN0000125104: CCGGGCAAAGTTATAGTGTCAGTTTCTCGAGAAACTGACACTATAACTTTGCTTTTTG, TRCN0000125105: CCGGCCCTTCCACTACAGTTTGAATCTCGAGATTCAAACTGTAGTGGAAGGGTTTTTG, TRCN0000125107: CCGGCCGATGGCATTACAGTGGTATCTCGAGATACCACTGTAATGCCATCGGTTTTTG) or a non-targeting negative control shRNA (Sigma; SHC204: CCGGCGTGATCTTCACCGACAAGATCTCGAGATCTTGTCGGTGAAGATCTTTTT) at MOI = 2.1*10^7^ TU/ml. After 14 days, cells were fixed for 12 minutes using 4% paraformaldehyde and 1% saccharose in PBS (pH 7.4), washed with PBS, permeabilized with 0.25% Triton X-100 in PBS for 10 minutes and blocked for 1 h in 1% BSA in 0.25% Triton X-100 in PBS. Neurons were stained with chicken anti-MAP2 (Bio Connect, Cambridge, UK; 1:5,000), and VAMP (Synaptic Systems, Billerica, MA, USA; 1:1,000) overnight at 4 °C. Antigens were visualized using DyeLight 549 (Jackson; 1:400) and Alexa 488 (Invitrogen 1:400) incubated for 2 h at room temperature. Cultures were imaged at 40x using an Opera LX HCS instrument (PerkinElmer, Waltham, MA). Columbus software (PerkinElmer) was used to quantify neuron numbers and synapse densities (i.e., total VAMP-positive puncta on MAP2-positive dendrites divided by the total dendrite length measured). Statistical significance was determined by Student t-tests.

## Additional Information

**How to cite this article**: Gonzalez-Lozano, M. A. *et al*. Dynamics of the mouse brain cortical synaptic proteome during postnatal brain development. *Sci. Rep.* **6**, 35456; doi: 10.1038/srep35456 (2016).

## Supplementary Material

Supplementary Information

Supplementary Table S1

Supplementary Table S2

Supplementary Table S3

Supplementary Table S4

Supplementary Table S5

Supplementary Table S6

## Figures and Tables

**Table 1:** Proteins with high fold differences at P9 compared with P280.

Gene name	Protein name	P9/P280 (log2)	Fold difference	BETR
Fabp7	Fatty acid-binding protein, brain	3.33	10.09	0.000
Dcakd	Dephospho-CoA kinase domain-containing protein	2.50	5.67	0.000
Mbp	Myelin basic protein	−2.47	5.54	0.000
Marcksl1	MARCKS-related protein	2.45	5.45	0.000
Cxadr	Coxsackievirus and adenovirus receptor homolog	2.21	4.64	0.000
Plp1	Myelin proteolipid protein	−2.17	4.50	0.000
Gjc2	Gap junction gamma-2 protein	−2.16	4.45	0.001
Sdc3	Syndecan-3	2.06	4.18	0.000
Stmn2	Stathmin-2	2.06	4.16	0.000
Slc1a2	Excitatory amino acid transporter 2	−1.90	3.74	0.000
Camk2a	Calcium/calmodulin-dependent protein kinase type II subunit alpha	−1.80	3.47	0.000
Tppp	Tubulin polymerization-promoting protein	−1.79	3.45	0.000
Tesc	Calcineurin B homologous protein 3	1.79	3.45	0.001
Dcx	Neuronal migration protein doublecortin	1.75	3.36	0.000
Tubb5	Tubulin beta-5 chain	1.73	3.31	0.000
Dpysl3	Dihydropyrimidinase-related protein 3	1.69	3.23	0.000
Stxbp5l	Syntaxin-binding protein 5-like	−1.69	3.22	0.000
Map1b	Microtubule-associated protein 1B	1.68	3.20	0.000
Hrsp12	Ribonuclease UK114	−1.60	3.03	0.000
Dhcr7	7-dehydrocholesterol reductase	1.60	3.02	0.000
Plgrkt	Plasminogen receptor (KT)	1.59	3.02	0.000
Eef1a1	Elongation factor 1-alpha 1	1.56	2.95	0.000
Tuba1a	Tubulin alpha-1A chain	1.54	2.91	0.000
Pdxk	Pyridoxal kinase	−1.53	2.88	0.000
Lmnb1	Lamin-B1	1.51	2.85	0.000
Eno2	Gamma-enolase	−1.47	2.77	0.000
Tuba4a	Tubulin alpha-4A chain	−1.47	2.77	0.000
Dpysl5	Dihydropyrimidinase-related protein 5	1.45	2.73	0.000
Atat1	Alpha-tubulin N-acetyltransferase 1	1.43	2.70	0.000
Pacsin1	Protein kinase C and casein kinase substrate in neurons protein 1	−1.43	2.70	0.000
Hapln4	Hyaluronan and proteoglycan link protein 4	−1.42	2.68	0.000
Nav1	Neuron navigator 1	1.42	2.68	0.000
Gap43	Neuromodulin	1.42	2.67	0.000
Sncb	Beta-synuclein	−1.41	2.66	0.000
Syn1	Synapsin-1	−1.41	2.66	0.000
Atp1a1	Sodium/potassium-transporting ATPase subunit alpha-1	−1.41	2.66	0.000
Nrn1	Neuritin	−1.40	2.64	0.000
Kcna1	Potassium voltage-gated channel subfamily A member 1	−1.37	2.59	0.000
Bdh1	D-beta-hydroxybutyrate dehydrogenase, mitochondrial	1.37	2.58	0.000
Tubb3	Tubulin beta-3 chain	1.37	2.58	0.000
Slc17a7	Vesicular glutamate transporter 1	−1.34	2.54	0.000
Cat	Catalase	1.34	2.53	0.000
Hapln1	Hyaluronan and proteoglycan link protein 1	−1.34	2.53	0.000
Thy1	Thy-1 membrane glycoprotein	−1.32	2.50	0.000
Sv2b	Synaptic vesicle glycoprotein 2B	−1.31	2.48	0.000
Stx1b	Syntaxin-1B	−1.31	2.48	0.000

**Table 2:** Diseases and functional annotations from proteins that show significant differences between P9 and P280 predicted by Ingenuity Pathway Analysis (IPA).

Functional annotation	p-value	Predicted activation	Molecules
neurotransmission	3.06E-51	decreased	103
long-term potentiation	3.87E-39	decreased	69
learn	6.42E-31	decreased	81
cognition	3.20E-30	decreased	84
transport of synaptic vesicle	6.99E-25	decreased	29
**Diseases and Disorders annotation**	**p-value**	**Predicted activation**	**Molecules**
movement disorder	5.37E-61	increased	186
neurological signs	9.31E-38	decreased	113
seizure disorder	1.84E-30	increased	87
tremor	8.27E-20	increased	36
ataxia	3.40E-15	increased	43
