# Supplementary material for: Dynamics of the mouse brain cortical synaptic proteome during postnatal brain development
Source: Sci Rep. 2016 Oct 17;6:35456. doi: 10.1038/srep35456 (PMC5066275; doi:10.1038/srep35456)
Supplement: Supplementary Information [file srep35456-s1.doc]

**Dynamics of the mouse brain cortical synaptic proteome during postnatal brain development**

Miguel A. Gonzalez-Lozano, Patricia Klemmer, Titia Gebuis, Chopie Hassan, Pim van Nierop, Ronald E. van Kesteren, August B. Smit, Ka Wan Li


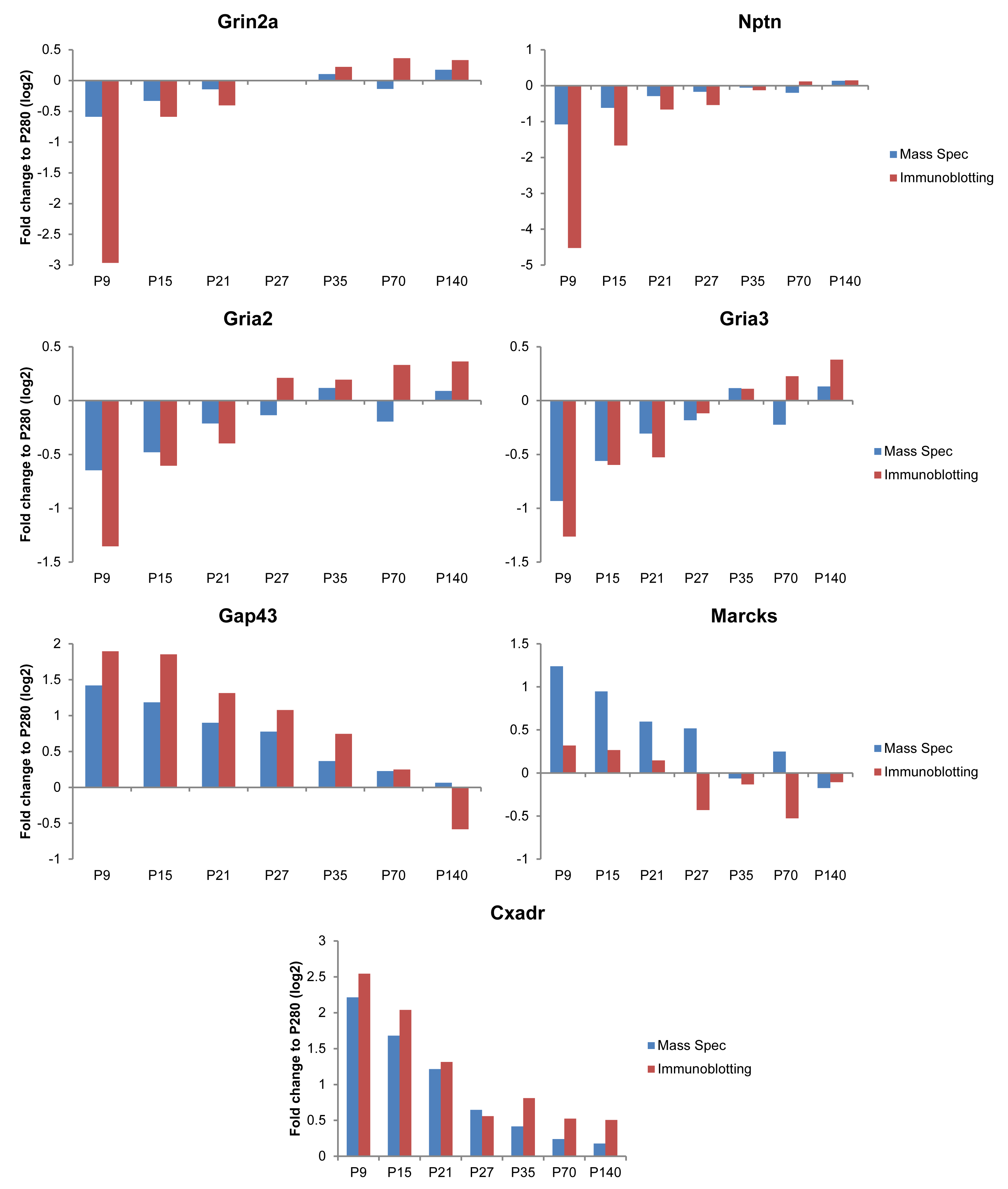


**Supplementary Fig. 1. Quantitative immunoblotting analysis of selected synaptic proteins.** The abundance profile of each protein depicted is presented as the ratio of signal intensity (fold difference on log2 scale) of the iTRAQ sets (blue) or immunoblotting (red) compared to the reference sample (P280). The *y*-axis indicates fold change on a log2 scale and changes in protein abundance with increasing age are shown from left to right (x-axis).
